# Representation of Early Sensory Experience in the Adult Auditory Midbrain: Implications for Vocal Learning

**DOI:** 10.1371/journal.pone.0061764

**Published:** 2013-04-24

**Authors:** Anne van der Kant, Sébastien Derégnaucourt, Manfred Gahr, Annemie Van der Linden, Colline Poirier

**Affiliations:** 1 Bio-Imaging Lab, University of Antwerp, Antwerp, Belgium; 2 Department of Behavioural Neurobiology, Max Planck Institute for Ornithology, Seewiesen, Germany; University of Texas at San Antonio, United States of America

## Abstract

Vocal learning in songbirds and humans occurs by imitation of adult vocalizations. In both groups, vocal learning includes a perceptual phase during which juveniles birds and infants memorize adult vocalizations. Despite intensive research, the neural mechanisms supporting this auditory memory are still poorly understood. The present functional MRI study demonstrates that in adult zebra finches, the right auditory midbrain nucleus responds selectively to the copied vocalizations. The selective signal is distinct from selectivity for the bird's own song and does not simply reflect acoustic differences between the stimuli. Furthermore, the amplitude of the selective signal is positively correlated with the strength of vocal learning, measured by the amount of song that experimental birds copied from the adult model. These results indicate that early sensory experience can generate a long-lasting memory trace in the auditory midbrain of songbirds that may support song learning.

## Introduction

Songbirds share with humans the ability to learn their vocalizations [1]–[3]. Like human babies need to be exposed to adult speech to develop a normal vocal repertoire, juvenile songbirds need to be exposed to adult conspecific vocalizations to develop a normal song (sensory phase). Then, during a subsequent sensori-motor phase, they use auditory feedback to progressively match their own developing vocalizations to the memorized adult model (called tutor song) [4]. Learning by imitation requires first to compare the motor performance with the object of imitation and then to correct for potential errors. It has long been hypothesized that the anterior forebrain pathway of songbirds, a circuit driving vocal variability in juveniles and adults [5]–[7], participates in both vocal error detection and error correction [8]. While the role of the anterior forebrain pathway in generating a corrective premotor bias has been recently confirmed [9], a growing number of studies point to the ascending auditory pathway as the main neural substrate of tutor song memory [10]–[15] and feedback-dependent error detection [16], [17]. However, if the auditory system supports the comparison between the bird's own song and a memory trace of the tutor song in order to detect vocal errors, one would expect to find bird's own song and tutor song selective signals in some of the auditory nuclei [18]. While significant bird's own song selective responses have been recently found in the auditory midbrain [19] and the auditory thalamus [17], evidence for tutor song selective responses in the ascending auditory pathway is still missing. The goal of this study was thus to look for tutor song selectivity in the auditory system, using blood oxygen level-dependent (BOLD) functional MRI (fMRI), a technique commonly used on humans and recently adapted to songbirds [20]. Such selectivity was found in the right auditory midbrain.

## Materials and Methods

### Ethical Statement

All experimental procedures were performed in accordance with the Belgian laws on the protection and welfare of animals and were approved by the ethical committee of the University of Antwerp, Belgium (EC nr 2009/21). All fMRI recordings were performed under isoflurane anesthesia and all efforts were made to minimize suffering and anxiety.

### Subjects

Twenty adult male (mean age 24 months, range 10–41 months) zebra finches (*Taeniopygia guttata*) recruited from the breeding colony of the Max Planck Institute for Ornithology (Seewiesen, Germany) were used in this experiment. Birds were raised by their parents from 0 to 7 days post hatching (DPH), by their mother from 8 to 34 DPH and were kept alone from 35 to 42 DPH. The birds were then housed singly with one adult male tutor from 43 to 100 DPH (one-to-one paradigm). Thirteen different tutors were used in the present experiment. These tutors previously learnt their own song from one of three song models via tape playback. Song data collected on the experimental birds and their tutors indicate that the three song models elicited similar amount of song copy. Following tutoring (after 100 DPH), the experimental birds were housed together, first in aviaries then in large cages. Birds were maintained throughout the experiment under a 12 h light∶12 h dark photoperiod and had access to food, water and baths *ad libitum*.

### Song Recording and Analysis

Prior to the fMRI experiment, each experimental bird was placed alone during 48 hours in a soundproof chamber and its song was recorded using the Sound Analysis Pro (SAP) 2.0 software ([21]; http://soundanalysispro.com/). Acoustic similarity between songs was assessed using the similarity score implemented in SAP. This measure is based on five acoustic features: pitch, frequency modulation, amplitude modulation, goodness of pitch and Wiener entropy and comprises two components: ‘the percentage of similarity’, measuring at a large scale (70 ms) the amount of sound shared between two songs and the ‘accuracy’, measuring the local, fine grained (10 ms) similarity (for more details, see SAP user manual, available at http://soundanalysispro.com/). The final score corresponds to the product of these two components. The computation of this similarity score was done by selecting one song as a reference (asymmetric measurement). To measure the vocal learning strength of each experimental bird, we selected the tutor song as the reference song, and compared the song of the tutee to this reference. This procedure was repeated 100 times, comparing 10 different exemplars of the tutor song with 10 different exemplars of the tutee song; the mean value was used. For measuring the acoustic similarity between stimuli used in the fMRI experiment (see below), there was no reason to choose one stimulus as a reference rather than the other one. For each pair of stimulus, we thus computed the similarity score twice, first using one stimulus of the pair as the reference, then using the other stimulus as the reference and finally computed the mean between the two indices.

### fMRI stimuli

**Figure 1:**
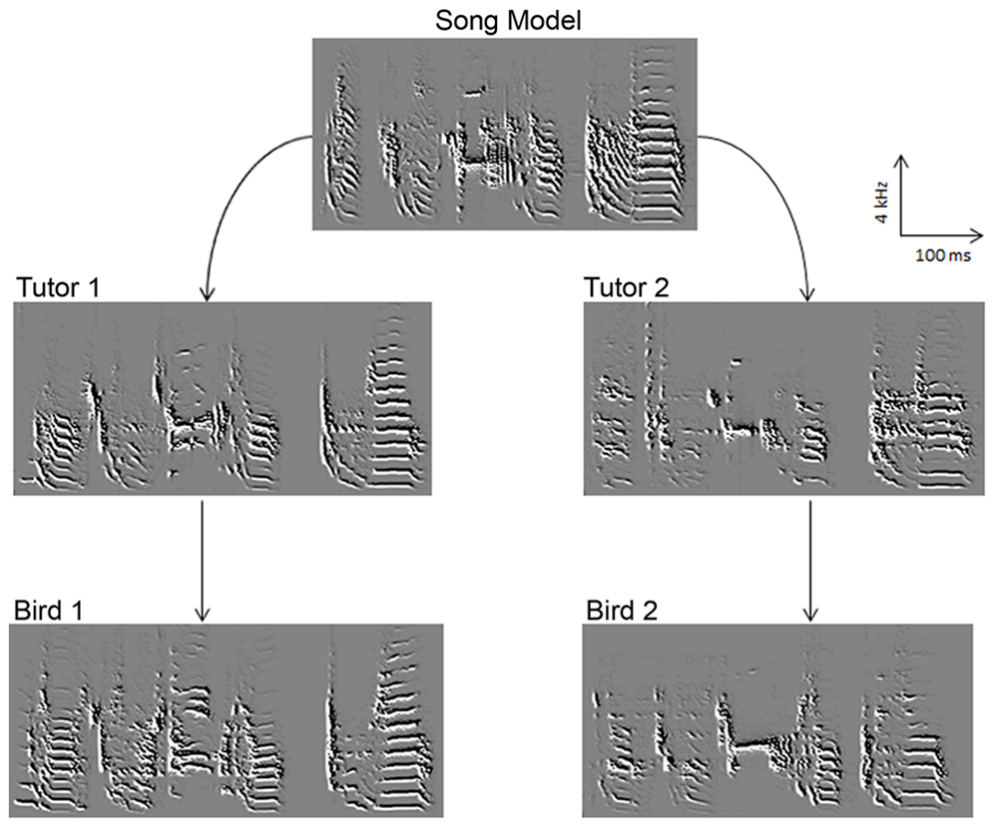
Sonograms illustrating the song tutoring protocol for two experimental birds (Bird 1 and Bird 2). Tutors 1 and 2 learned their song from the same song model (via tape playback) while experimental birds 1 and 2 learned their song by being housed with respectively tutor 1 and tutor 2 (one-to-one paradigm). As a result, songs of Bird 1 and 2 were acoustically close. During the fMRI experiment, bird 1 was exposed to the song of bird 1 (BOS), the song of Tutor 1 (TUT) and the song of Bird 2 (CON).

For each experimental bird, three familiar songs were used as stimuli in the fMRI experiment: the bird's own song (BOS), the tutor song (TUT) and a conspecific song (CON). The conspecific song came from an adult bird housed during several weeks in the same aviary/cage as the experimental bird after the end of the learning phase (i.e. after 100 DPH). This adult bird had been previously raised by a tutor, which had learnt to copy the same song model than the tutor of the experimental bird (fig. 1). As a result, the CON stimulus was thus not only familiar to the experimental bird but also acoustically close to its own song and its tutor song. For each bird, stimuli corresponded to one song exemplar of each category (BOS, TUT and CON), picked up randomly from the 10 exemplars used for computing the learning strength value (see above). Measures of acoustic similarity revealed no significant difference between the three stimuli (Repeated measure one-way ANOVA: F = 0.98, p = 0.39). Post-hoc paired t-tests confirmed the absence of significant difference between each pair of stimulus (TUT/CON similarity vs. TUT/BOS similarity: t = 0.48, p = 0.64; TUT/CON similarity vs. BOS/CON similarity: t = 1.3, *p* = 0.21; TUT/BOS similarity vs. BOS/CON similarity: t = 1.1, *p* = 0.28).

### Experimental setup and design

During the experiment, birds were continuously anaesthetized with 1.5% isoflurane. Auditory stimuli were played back at a mean intensity (in term of Root Mean Square) of 70 dB through small loudspeakers (Visation, Germany) from which magnets were removed. An equalizer function was applied to the stimuli using WaveLab software (Steinberg, Germany) to correct for enhancement of frequencies between 2500 and 5000 Hz in the magnet bore (see Poirier *et al*, 2010). Stimulus delivery was controlled by Presentation 0.76 software (Neurobehavioral Systems Inc., Albany, CA, USA).

During fMRI acquisition, the three stimuli were randomly presented in an ON/OFF blocked design where 16 s stimulation (ON blocks) and 16 s rest periods (OFF blocks) were alternated. Each ON block included repetitions of the same stimulus interleaved with silent periods. The duration of the silent periods was adjusted in each bird to match the amount of song and silence between stimuli (mean song duration: 11.2 s for each stimulus; mean silence duration: 4.8 s). The experiment consisted in 93 ON blocks (31 per stimulus) and 93 OFF blocks. During each block, 2 magnetic resonance images were acquired, resulting in 62 images per stimulus and per subject.

### fMRI acquisition

BOLD fMRI images were acquired using a 7T Pharmascan system (Bruker, Erlangen, Germany). Details about this system and the coils used for the experiment can be found in [22]. For each bird, a time series of 372 T_2_-weighted rapid acquisition relaxation-enhanced (RARE) Spin Echo (SE) images (Effective Echo time (TE)/Repetition time (TR): 60/2000 ms; RARE factor: 8; Field of View: 16×16 mm) was acquired. Images comprised 15 slices (in-plane resolution: 250×250 µm^2^) with a slice thickness of 750 µm and an inter-slice gap of 50 µm, covering the whole brain. Following the fMRI acquisition, a high-resolution anatomical three-dimensional (3D) SE RARE image (voxel size 125 µm^3^; TE/TR: 60/2000 ms; RARE factor: 8; Field of View: 16×16 mm) was acquired for each bird.

### Image processing

Data processing was carried out using SPM8 (Wellcome Trust Centre for Neuroimaging, London, UK; http://www.fil.ion.ucl.ac.uk/spm/). To enable an accurate localization of the functional activations, the high-resolution anatomical 3D images of each subject were normalized to the MRI atlas of the zebra finch brain [23]. Each fMRI time series was realigned to correct for head movements, co-registered to the high-resolution 3D image of the same bird and up-sampled to obtain a resolution of 125×125×400 µm, as classically done in fMRI data processing. These steps resulted in a good correspondence between the fMRI data and the anatomical data from the atlas. Finally, the fMRI images were smoothed with a Gaussian kernel (width of 500×500×800 µm^3^).

### Statistical analysis

**Figure 2:**
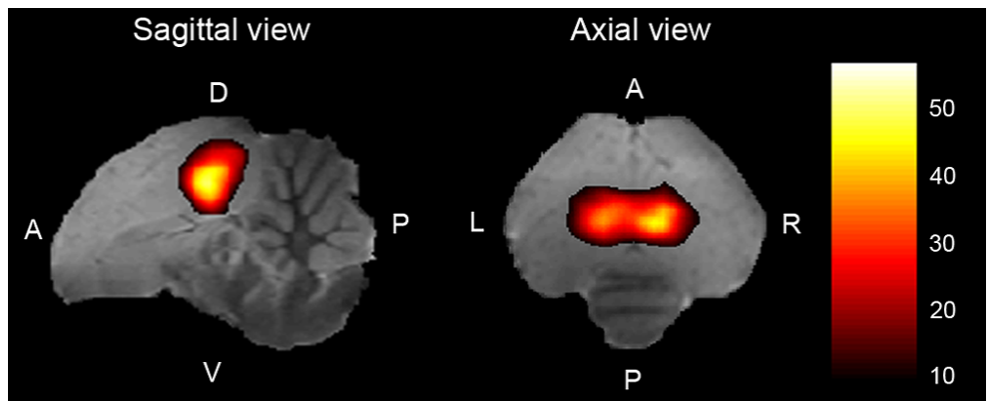
Statistical maps of BOLD activation induced by all stimuli together. Results (compared to Rest) are superimposed on anatomical sagittal and axial images coming from the MRI zebra finch atlas. T values are color coded according to the scale displayed on the right side of the figure. Only significant voxels (one-tailed t-test, p<0.05, corrected at the whole brain level) are displayed. L: left, R: right, D: dorsal, V: ventral, A: anterior, P: posterior.

Statistical analysis of the fMRI data was performed at the subject and group level in SPM8, using the General Linear Model. Data were modeled as a box-car and filtered with a high-pass filter of 352 sec. Model parameters were then estimated using a classical restricted maximum likelihood algorithm. Subject-level analyses were performed to identify the mean effect [All stimuli minus rest] in each individual subject. These analyses revealed a bilateral positive BOLD signal in the auditory telencephalic regions (fig. 2) of 17 birds over 20, a success rate similar to the one obtained in our previous spin-echo fMRI experiments [19], [24], [25]. A bilateral response to the stimulation paradigm in the auditory regions confirms that the stimulation has been processed by the auditory system and was therefore used as an inclusion criterion. The subsequent analyses were thus only performed on these 17 birds, data from the 3 remaining birds being discarded.

**Figure 3:**
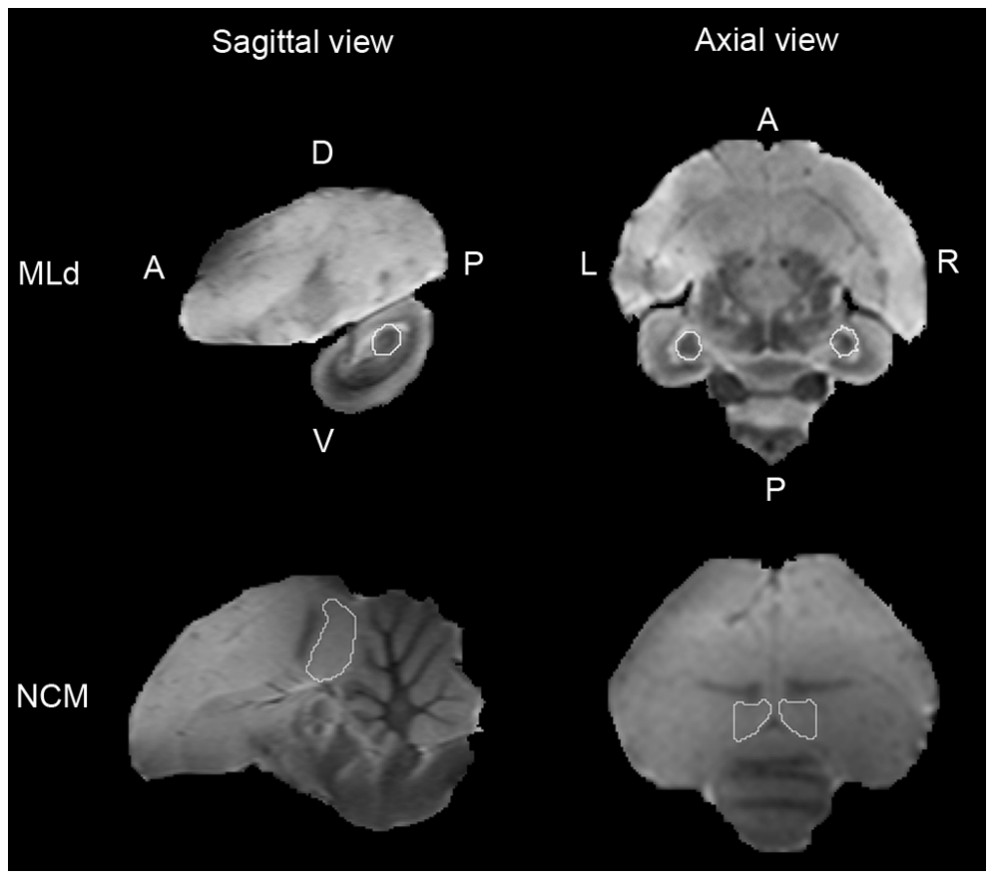
Illustration of the predefined regions of interest on sagittal and axial anatomical images. The anatomical images come from the zebra finch MRI atlas. L: left, R: right, D: dorsal, V: ventral, A: anterior, P: posterior.

The effect of [each stimulus minus rest] of each subject was then entered in a group-level random effect analysis. The mean effect [All stimuli minus rest] at the group level revealed a positive BOLD response not only in the auditory telencephalic regions but also in the dorsal part of the lateral mesencephalic nucleus (MLd), the main auditory midbrain nucleus. In order to increase the sensitivity of the statistical analyses, we focused on two pre-defined regions of interest in each hemisphere: MLd, where bird's own song selectivity has been previously found [19] and the caudomedial nidopallium (NCM) (fig. 3), a telencephalic auditory region involved in tutor song memory [10]–[12], [14], [15]. MLd could be clearly identified and delineated on the zebra finch atlas [21]. NCM was delineated using Field L as anterior border, the cerebellum as posterior border and the lateral ventricle as ventral and dorsal borders. The lateral boundaries of NCM are not defined from a cyto-architectural point of view. In accordance with previous functional studies [26]–[30], we included the three 0.4 mm-thick slices covering brain tissues between 0.2 mm and 1.4 mm from the midline in each hemisphere.

Statistical differences between stimulus-evoked BOLD signals were assessed in each voxel of the predefined regions using a one-way repeated measure ANOVA (F-tests) followed by post-hoc one-tailed paired t-tests. P values were corrected for multiple tests using the Family Wise Error method based on the Random Field Theory [31]. In addition, an extent threshold was applied to the results: activations had to consist of a cluster of at least 5 significant contiguous voxels (corrected p value<0.05) to be considered statistically significant. Reflecting the voxel basis of the analysis, results are reported by the highest voxel F/t value within each cluster (F_max_/t_max_) and the associated voxel p value. Regression analyses were also performed to assess potential correlations between the amplitude of differential fMRI signals ([BOS minus CON] and [TUT minus CON]) and various behavioral measures. In MLd, these analyses were performed by taking the mean fMRI signal averaged over the contiguous voxels in which a significant differential fMRI signal was first demonstrated. When applied to a brain region which can be reasonably assumed to be homogeneous, this procedure is more representative of data than a voxel-based analysis (i.e. correlation analysis performed in each individual significant voxel). Note however that a voxel-based analysis has also been performed and provided similar results (not described in the present manuscript). In NCM, because the main effect of the ANOVA did not yield significant results, a correlation analysis between non-significant differential fMRI signals and learning strength was not meaningful. However, because previous authors reported a correlation between TUT-induced immediate early gene expression and learning strength in NCM [26]–[28], we tested for potential correlation between [TUT minus Rest] and learning strength. Here, because the comparison [TUT minus Rest] was found significant in most part of NCM, we used a voxel-based approach. This approach was considered more relevant than using the mean fMRI signal averaged over all the NCM contiguous significant voxels because of the big size of NCM and the numerous studies suggesting that NCM comprises anatomically and functionally different sub-regions (e.g. [30], [32], [33]). Subsequent correlation analyses between learning strength and respectively [BOS minus Rest] and [CON minus Rest] were then limited to the small part of NCM where a correlation between [TUT minus Rest] and learning strength had been found, and were performed on the mean fMRI signal averaged over the contiguous voxels of this small region.

## Results

### Behavioral results of song tutoring

On average, the one-to-one tutoring protocol induced significant learning of the tutor song from the tutees: the mean learning strength, measured by the SAP similarity score including large-scale and fine-grained similarity, was of 48% (SE = 3.2), whereas the similarity of the tutee song with songs of other experimental birds heard only after what is supposed to be the end of the learning period (100 DPH) was of 28% (SE = 1.5). When learning strength was assessed by the SAP similarity score restricted to large-scale similarity, the mean value was 67%, which is within the range of what is accepted as normal tutor song copy; for instance, birds trained with tape recordings of adult songs were previously reported to have a large-scale SAP similarity score of 61% while birds raised with their parents had a score of 71% [11].

### Brain responses in MLd

**Figure 4:**
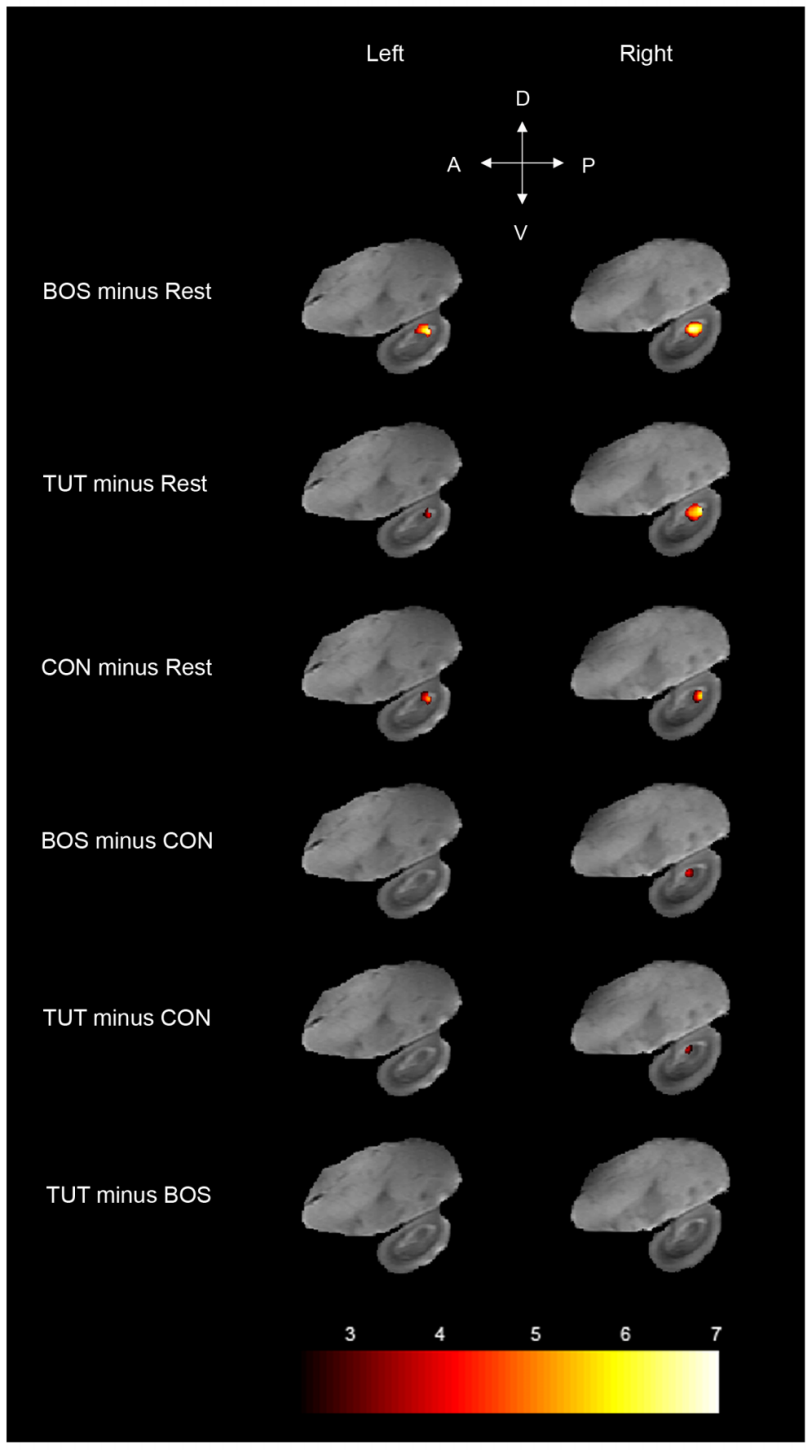
Statistical maps of BOLD activation induced by the different stimuli in left and right MLd. Results are superimposed on sagittal anatomical slices coming from the MRI zebra finch atlas. T values are color coded according to the scale displayed at the bottom of the figure. Note that the analysis was restricted to MLd and only voxels found to be significant (one-tailed t-test, p<0.05, corrected at MLd level) are displayed. D: dorsal, V: ventral, A: anterior, P: posterior.

Right and left MLd were significantly positively activated by the three song stimuli BOS, TUT, and CON (Fig. 4; Left MLd: [BOS minus Rest]: t_max_ = 6.7, *p*<0.0001; [TUT minus Rest]: t_max_ = 4.5, *p* = 0.001; [CON minus Rest]: t_max_ = 5.2, *p* = 0.0001; Right MLd: [BOS minus Rest]: t_max_ = 6.9, *p*<0.0001; [TUT minus Rest]: t_max_ = 6.7, *p*<0.0001; [CON minus Rest]: t_max_ = 6.0, *p*<0.0001). Significant differences in term of BOLD response amplitude elicited by different stimuli were found in right MLd (F_max_ = 10.3, *p* = 0.01) but not in left MLd (F_max_ = 3.2, *p* = 0.35). Post-hoc paired t-tests in right MLd revealed that the main effect was due to a greater activation induced by BOS and TUT compared to CON ([TUT minus CON]: t_max_ = 4.1, *p* = 0.005; [BOS minus CON]: t_max_ = 4.0, *p* = 0.005; [TUT minus BOS]: t_max_ = 1.1, *p* = 0.57).

**Figure 5:**
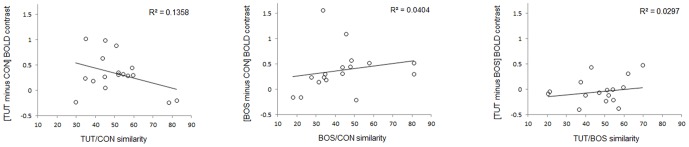
Correlation between MRI signals and the acoustic similarity between the stimuli in right MLd. The MRI signals (expressed in non-dimensional units) correspond to the mean amplitude estimate of the differential BOLD signals between TUT and CON (left), BOS and CON (middle) and TUT and BOS (right). Positive values on the y axis indicate higher activations induced by the first stimulus of the comparison than the second one while negative values indicate higher activations induced by the second stimulus of the comparison than the first one. All correlations are statistically non-significant.

Besides the fact that the mean acoustic similarity was not significantly different between each pair of stimuli (see Materials and Methods), we further examined whether the amplitude of the differential activations was correlated with the acoustic similarity between the stimuli. None of the correlations was significant (Fig. 5; [TUT minus CON] vs. TUT/CON similarity: R^2^ = 0.14, p = 0.15; [BOS minus CON] vs. BOS/CON similarity: R^2^ = 0.04, p = 0.44; [TUT minus BOS] vs. TUT/BOS similarity: R^2^ = 0.03, p = 0.51), excluding the acoustic similarity between the stimuli as the mere explanation for the amplitude of the differential activations.

**Figure 6:**
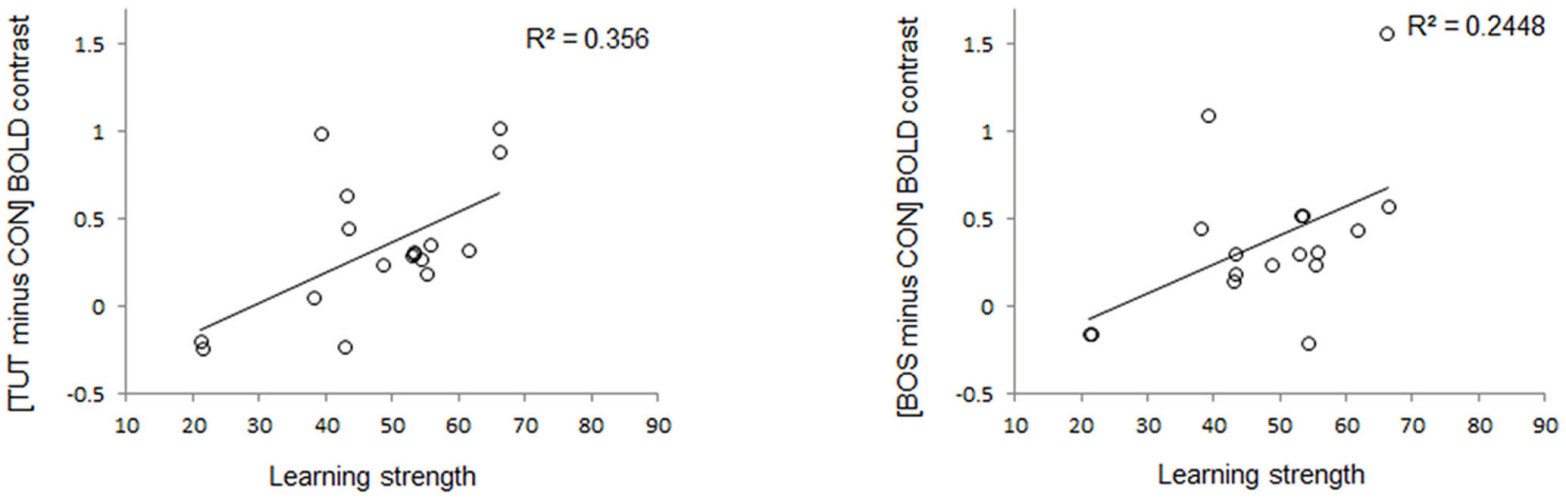
Correlation of TUT (left) and BOS (right) selectivity with vocal learning strength in right MLd. TUT and BOS selectivity are expressed as the mean amplitude estimate of the differential BOLD signals of [TUT minus CON], and [BOS minus CON], in non-dimensional units. Positive values on the y axis indicate a higher activation induced by TUT (or BOS) compared to CON while negative values indicate a higher activation induced by CON compared to TUT (or BOS). Both correlations are statistically significant.

We then looked whether the amplitude of the TUT and BOS selective signals (defined respectively as [TUT minus CON] and [BOS minus CON] BOLD responses) could reflect the amount of sound each experimental bird copied from its tutor (learning strength). This analysis revealed a significant positive correlation between TUT selectivity and learning strength (Fig. 6; R^2^ = 0.36, *p* = 0.01) as well as between BOS selectivity and learning strength (R^2^ = 0.25, *p* = 0.04).

Finally, we tested for potential correlations between the amplitude of BOS and TUT selectivity and the age of birds. The two correlations were non-significant ([TUT minus CON]: R^2^<0.01, *p* = 0.80, [BOS minus CON]: R^2^<0.01, *p* = 0.78).

### Brain responses in NCM

**Figure 7:**
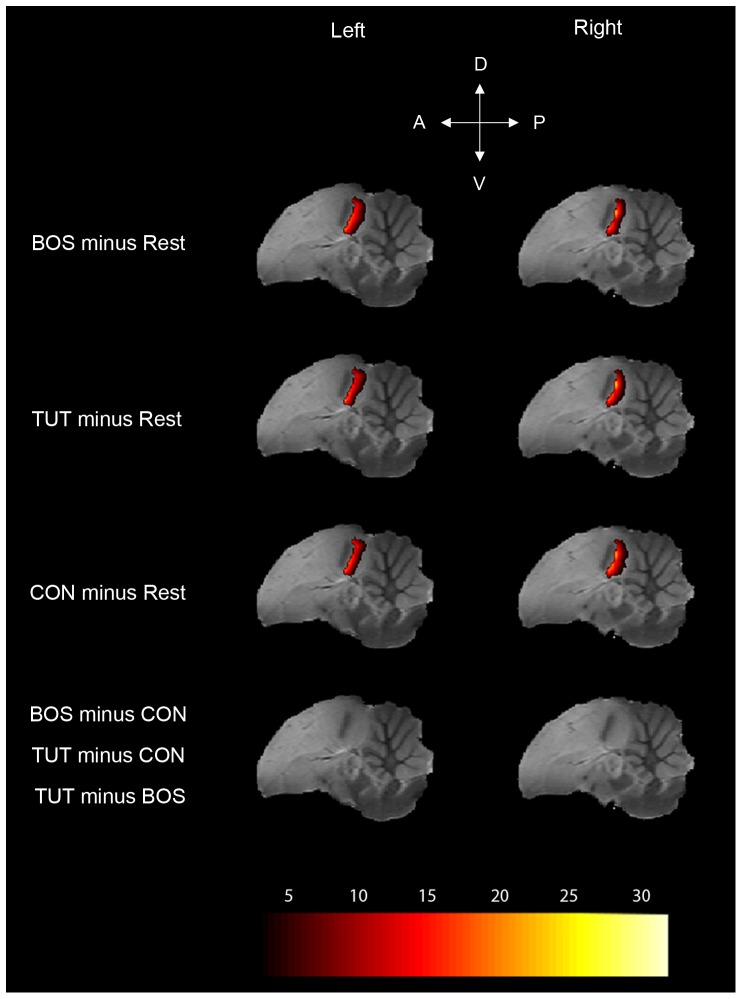
Statistical maps of BOLD activation induced by the different stimuli in left and right NCM. Results are superimposed on sagittal anatomical slices coming from the zebra finch MRI atlas. T values are color coded according to the scale displayed at the bottom of the figure. Note that in the figure other auditory regions (Field L and caudo-medial mesopallium) seem not activated only because the statistical analysis was restricted to NCM (for the whole activation pattern in the telencephalic auditory regions, see fig. 2). Only significant voxels (one-tailed t-test, p<0.05, corrected at NCM level) are displayed. D: dorsal, V: ventral, A: anterior, P: posterior.

Left and right NCM were significantly positively activated by the three stimuli (Fig. 7; Left NCM: [BOS minus Rest]: t_max_ = 22.3, *p*<0.0001; [TUT minus Rest]: t_max_ = 22.2, *p*<0.0001; [CON minus Rest]: t_max_ = 22.4, *p*<0.0001; Right NCM: [BOS minus Rest]: t_max_ = 32.2, *p*<0.0001; [TUT minus Rest]: t_max_ = 33.9, *p*<0.0001; [CON minus Rest]: t_max_ = 33.1, *p*<0.0001). We did not find any significant difference in term of BOLD response amplitude between the stimuli (Left NCM: F_max_ = 3.0, *p* = 0.88; Right NCM: F_max_ = 4.4, *p* = 0.65).

**Figure 8:**
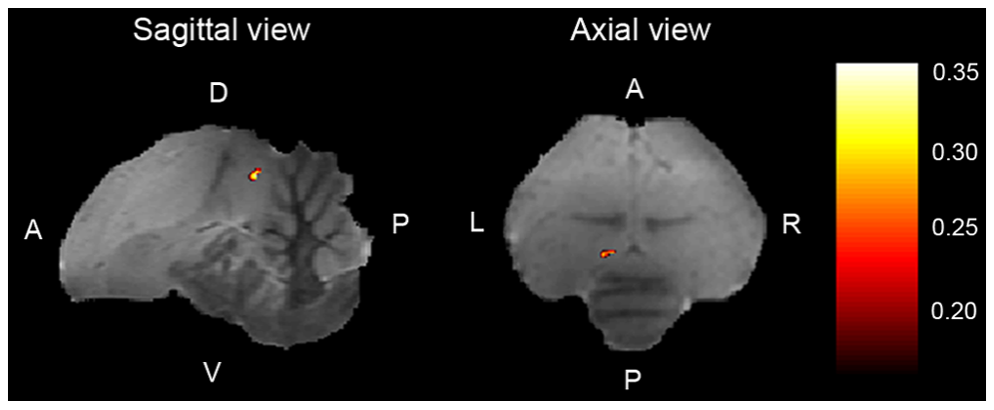
Correlation map of [TUT minus Rest] versus vocal learning strength in left NCM. Results are superimposed on sagittal and axial anatomical slices coming from the zebra finch MRI atlas and displayed at a p threshold of 0.05 without correction for multiple tests. R^2^ values are color coded according to the scale displayed at the right side of the figure. D: dorsal, V: ventral, A: anterior, P: posterior; L: left; R: right.

**Figure 9:**
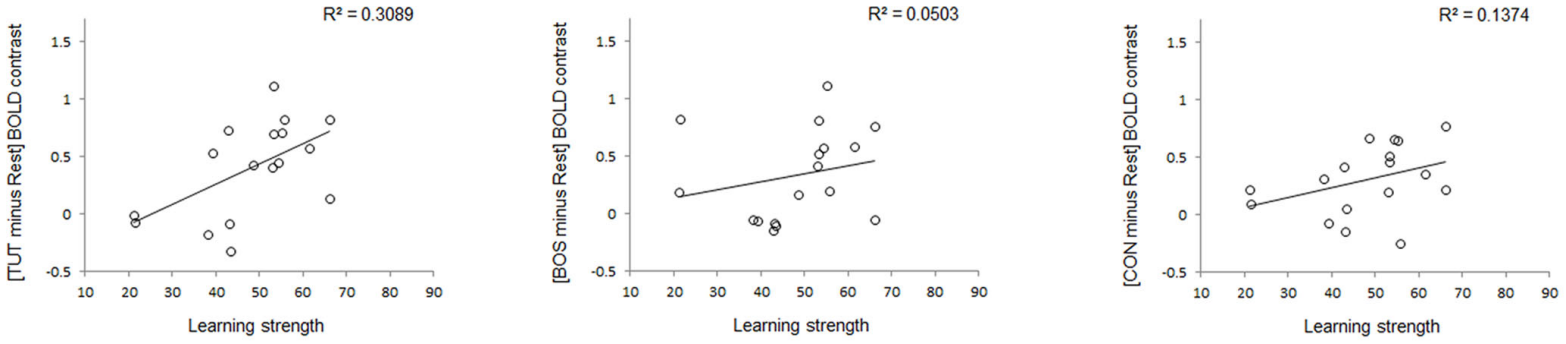
Correlation of TUT, BOS and CON responsiveness with vocal learning strength in left NCM. TUT, BOS and CON responsiveness are expressed as the mean amplitude estimates of the BOLD activations [TUT minus Rest], [BOS minus Rest] and [CON minus Rest], in non-dimensional unit) in the left NCM cluster illustrated in Fig. 8. Note that the R^2^ value in the left panel (0.3089) corresponds to the correlation value between learning strength and the [TUT minus Rest] signal averaged over the NCM cluster illustrated in Fig. 8 whereas the value reported in the text (0.36) corresponds to the correlation in the voxel where this correlation is the highest (R^2^_max_). These two R^2^ values are significantly different than 0. Correlation of BOS and CON responsiveness with learning strength are not significant.

The lack of significant differential activation in NCM prevented us to test for potential correlation between differential activations and learning strength. Nevertheless, a correlation between [TUT minus Rest] and learning strength could be expected in NCM based on earlier studies [28]–[30]. Such analysis failed to reveal any significant correlation (left NCM: R^2^_max_ = 0.36, *p* = 0.15, Right NCM: R^2^_max_ = 0.09, *p* = 0.86). However one can notice that the maximal correlation value measured in left NCM was of the same magnitude as the one measured between TUT selectivity and learning strength in right MLd (R^2^ = 0.36 for both correlations). The big difference in term of p values is due to the correction for multiple tests applied in NCM (corrected/uncorrected p value = 0.15/0.006), which is directly related to the size of the investigated region. The correlation analyses performed on NCM were thus much less sensitive than the ones performed on MLd. Interestingly, a cluster of voxels in left NCM surviving the uncorrected p threshold of 0.05 was located in the posterior and lateral part of NCM (fig. 8), where Bolhuis and colleagues previously found a significant correlation between tutor song evoked gene expression and learning strength [28]–[30]. Intrigued by this similitude, we further explored whether the correlation with learning strength was specific to tutor song or whether similar results could be found for BOS and CON evoked activations. These last analyses revealed no correlation of learning strength with [BOS minus Rest] and [CON minus Rest] (fig. 9, R^2^<0.14; p values>0.14), suggesting that as in Terpstra et al. study [30], the correlation was specific to the tutor song.

## Discussion

The present study demonstrates selectivity for tutor song and bird's own song in right MLd, the main auditory midbrain nucleus. This selectivity was defined by a higher BOLD response induced by TUT and BOS than by CON. The impact of acoustic features was controlled by using a conspecific song acoustically close to BOS and TUT and by *a posteriori* testing potential correlation between the strength of selective signals and the estimated amplitude of the residual acoustic differences between the stimuli. Such correlations were found not significant, ruling out the acoustic parameters as the main experimental factor responsible for the selectivity. This result rather suggests that it is the interaction between the acoustic features and the stimulus history which is responsible for the selectivity. The nature of the stimulus history responsible for the selectivity can be narrowed down since we used a familiar conspecific song as a control stimulus. The conspecific song came from a bird housed with the experimental bird after the end of the sensori-motor learning period (i.e. after 100 DPH), indicating that selective signals were induced by songs learned during the sensory-motor learning period.

Since the tutor song and the bird's own are usually acoustically close, it has been suggested that responses to the tutor song might reflect sensitivity to the bird's own song [34]. In the present study, BOS and TUT stimuli induced BOLD responses of similar amplitude. However, if the acoustic similarity was responsible for this lack of significant difference, similar BOLD responses should have been also found between BOS and CON since the acoustic similarity was not significantly different between each pair of stimuli. On the contrary, BOS and CON induced neural responses of significantly different amplitude. One would also expect the difference between BOS and TUT BOLD responses to be negatively correlated with the acoustic similarity between the two stimuli, which was not the case in the present study. Altogether, these results indicate that the right MLd is selective for both stimuli. BOLD fMRI signal reflects the activity of large populations of neurons. It is thus possible that different neuronal sub-populations are selective for the bird's own song and the tutor song. Alternatively, the same neurons could be selective for the two types of stimuli, as it has been shown in few neurons of the anterior forebrain pathway [35].

The tutor song selectivity found in the right auditory midbrain indicates that a representation of the tutor song is still present in the adult brain. Since the tutor song is the song memorized by the experimental bird and later used to guide its vocal practice, the presence of selective responses which cannot be explained by acoustic differences between the stimuli strongly suggest that MLd is part of the neural substrates of tutor song memory. Reinforcing this interpretation, the strength of TUT selectivity was found to be positively correlated with the amount of song that the experimental birds copied from their tutor. This correlation suggests that birds that formed an accurate or well-consolidated memory of their tutor' song later produced an accurate copy of this song.

BOS selectivity in right MLd constitutes an important replication of our previous findings [19]. The present study demonstrates that this selectivity can be detected even when the conspecific song used as a control stimulus is acoustically close to the bird's own song. Birdsong is thought to be learned by trial and error. Detecting vocal errors supposes to identify the current state of the bird's own song via the auditory feedback, and then to compare it with the memorized tutor song. Bird's own song selective responses are thought to support these mechanisms [36], [37]. Bird's own song selectivity in right MLd could thus reflect the identification of the bird's own song current state or the output of the comparison between the current song and the tutor song memory. The strength of bird's own song selectivity in MLd was found positively correlated with the amount of song experimental birds copied from their tutor. This result might suggest that bird's own song selectivity reflects the output of the comparison, the selective signal being stronger when the current song is found closer to the tutor song memory. Alternatively, this correlation could reflect the accuracy of bird's own song current state identification: indeed, an accurate bird's own song encoding is necessary to produce an accurate copy of the tutor song. Since tutor song selective responses were also found in the same nucleus, the subsequent comparison of the current bird's own song with the tutor song memory could then be made in MLd main efferent target, the auditory nucleus of the thalamus, and/or downstream, in the telencephalic auditory regions. This hypothesis is supported by recent evidence indicating that neurons in these thalamic and telencephalic regions increase their activity in response to feedback perturbations and thus could encode information about the quality of the bird's own song relative to the tutor song [16], [17].

**Figure 10:**
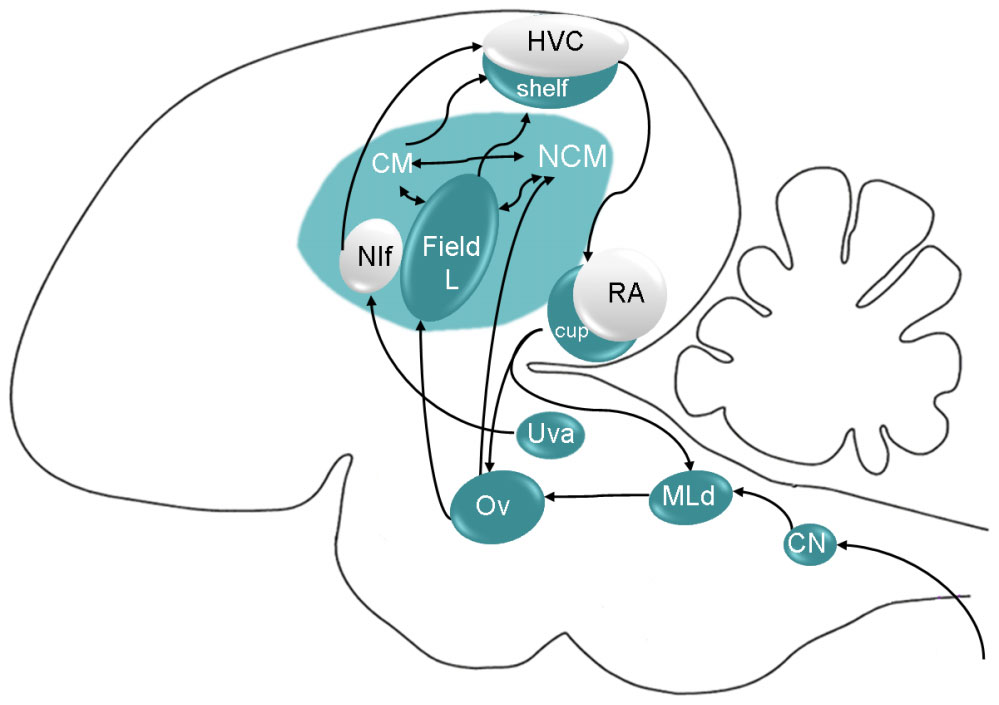
Schematic representation of the songbird brain (parasagittal view). The auditory regions are in blue and the vocal motor regions in grey. Only the main connections are represented. NIf: nucleus interface of the nidopallium; Ov: nucleus ovoidalis; RA: nucleus robustus of the arcopallium; Uva: nucleus uvaeformis; CN: cochlear nucleus.

Numerous studies have pointed to another region of the ascending auditory pathway, NCM, to be involved in tutor song memory [10]–[15]. One of these studies has shown that despite a similar amount of immediate early gene expression evoked by the tutor song, the bird's own song and a novel song in the lateral part of NCM of adult birds, only the activity evoked by the tutor song was positively correlated with the quality of tutor song imitation [30]. A similar trend was observed in the present fMRI study. In the ascending auditory pathway, MLd sends projection to the auditory nucleus of the thalamus called Ovoidalis, which projects to Field L at the telencephalic level (fig. 10). Field L then projects to NCM and the caudal mesopallium (CM). Along this pathway, the information is considered to be encoded in a hierarchical way, neurons in NCM and CM being more complex than those in MLd (for a recent review, see [38]). For instance MLd is known to respond to a wide variety of sounds, including conspecific and heterospecific songs but also tones and white noise while NCM mainly responds to conspecific songs. MLd neuronal responses are also more reliable, encoding precisely the spectro-temporal characteristics of the stimuli and are less context-dependent than NCM responses. While our results are consistent with recent evidence showing that MLd neurons can encode the identity of individual songs [39] and that their activity can be modulated by early auditory experience [40], the fact that tutor and bird's own song selectivity was found in the MLd of adult birds and not in NCM does not fit well with a hierarchical organization. We cannot rule out that the lack of selectivity in NCM is not due to the limited sensitivity of our experiment. Alternatively, the fact that the correlation of neural activity with learning strength was associated with selectivity for the tutor song in MLd but not in NCM suggests that the two regions play different roles putatively supported by different underlying mechanisms and different neural pathways. It has been recently demonstrated that the nucleus interface of the nidopallium and HVC (used as a proper name), two pre-motor nuclei displaying bird's own song selective responses, play an important role in tutor song encoding [41]. The nucleus ovoidalis is suspected to send projections to the nucleus interface of the nidopallium [42], which projects to HVC. MLd selective responses could thus reflect activity in this alternative pathway. Finally, the shelf of HVC sends projection to the area surrounding the nucleus robustus of the arcopallium which projects to Ovoidalis and MLd (fig. 10). Our results might thus reflect activity in these descending projections.

MLd tutor song and bird's own song selective signals described in the present study have been detected in anesthetized birds. A recent report indicates that tuning properties of MLd neurons are similar in awake and anesthetised individuals [43]. Additionally, results of the present experiment in NCM constitute a replication of what have been found with another technique in awake birds [30], suggesting that anesthesia did not have a strong influence on the results. On the other hand, bird's own song selective responses in other forebrain regions have been found to be present when birds are anesthetised or asleep but to vanish when birds are alert [44], [45]. Because these selective responses mimic spontaneous on-going activity occurring during sleep, they have been interpreted as reflecting off-line memory consolidation processes [46]. Playback of tutor song during the day has also been found to induce in juvenile birds specific changes in bursting activity of neurons during the following night of sleep, suggesting again that memory consolidation processes took place during the night [47]. Tutor song and bird's own song selective signals found in MLd might thus alternatively reflect such off-line memory consolidation processes. Either way (on-line or off-line mechanisms), the behavioural relevance of MLd selective signals in term of song learning is supported by the correlation found between the strength of the selectivity and the amount of song juvenile birds copied from their tutor.

Finally, bird's own song and tutor song selectivity was found in right but not left MLd. Even if investigating the lateralization of the responses was beyond the scope of this study, these results comfort the right lateralization of bird's own song selective responses found in MLd in our previous study [19]. A recent study suggests that lateralization for conspecific song at the telencephalic level depends on auditory experience [48]. At the midbrain level, auditory experience has been shown to influence information coding and firing rate of MLd neurons [40]. Whether lateralization of MLd responses is also experience-dependent should be the object of further investigation.

To conclude, this study indicates that a memory trace of the vocalizations used as a model to guide vocal learning is present in the right auditory midbrain of adult songbirds. By showing that early sensory experience can generate long-lasting memories in a brainstem structure, it provides additional evidence to the growing body of research showing that that experience-dependent plasticity is not limited to cortical structures [49], [50]. Recent studies indicate that the human auditory brainstem is involved in foreign language learning [51], [52] and training-based improvement of speech hearing in noise [53] in adults. Since the organization of the auditory pathway at the sub-cortical level is well conserved among vertebrates, the involvement of the auditory midbrain in the auditory memory supporting vocal learning might be important for both avian and mammalian vocal learners.
